# Chronic Myeloid Leukemia Harboring a Novel Four-Way Variant Translocation t(7;22;9;15)(p15;q11.2;q34.1;q22) With Cryptic Double Philadelphia Chromosomes

**DOI:** 10.7759/cureus.95633

**Published:** 2025-10-28

**Authors:** Shogo Kamigaki, Makoto Ito, Satsuki Nakano, Norio Takagi

**Affiliations:** 1 Department of Hematology, Chitahanto Rinku Hospital, Tokoname, JPN; 2 Department of Hematology and Oncology, Nagoya University, Nagoya, JPN; 3 Department of Pathology, Chitahanto Rinku Hospital, Tokoname, JPN; 4 Department of Pathology and Molecular Diagnostics, Nagoya City University Graduate School of Medical Sciences, Nagoya, JPN

**Keywords:** chronic myeloid leukemia (cml), cytogenetics, dasatinib, double philadelphia chromosome, four-way translocation

## Abstract

Variant Philadelphia chromosomes are occasionally observed in chronic myeloid leukemia. Among them, four-way variant Philadelphia translocations are particularly rare. Double Philadelphia chromosomes, representing an additional Philadelphia chromosome, are also uncommon and have been reported to be associated with disease progression and therapeutic challenges. We report a case of chronic myeloid leukemia with a novel four-way variant translocation t(7;22;9;15)(p15;q11.2;q34.1;q22) accompanied by cryptic double Philadelphia chromosomes. Despite these unfavorable cytogenetic features, the patient was successfully treated with dasatinib and achieved a complete hematologic response.

## Introduction

Chronic myeloid leukemia (CML) is characterized by the presence of the Philadelphia chromosome (Ph), which results from a reciprocal translocation between chromosomes 9 and 22, leading to the fusion of the BCR and ABL genes [1]. The advent of tyrosine kinase inhibitors (TKIs) targeting BCR-ABL1 has dramatically improved patient outcomes, transforming CML from a fatal disease into a manageable chronic condition for most patients [2].

Variant Ph chromosomes, which involve additional chromosomes beyond the classic t(9;22)(q34;q11), are observed in approximately 5-10% of CML cases [3]. Their clinical significance remains a matter of investigation, with some studies suggesting no major impact on prognosis, while others report associations with treatment resistance or disease progression [3-6]. Among these, four-way (4-way) variant Ph translocations are particularly rare, with approximately 80 cases documented to date [4].

Double Ph chromosomes, characterized by an additional Ph chromosome beyond the standard single Ph, are uncommon at initial diagnosis in chronic-phase (CP) CML (<5%) but increase substantially, up to 30-40%, during progression to accelerated phase (AP) or blast crisis (BC) [6,7]. The presence of double Ph chromosomes has been reported to correlate with clonal evolution, higher leukemic burden, and reduced sensitivity to first-generation TKIs such as imatinib (IMA), thereby necessitating alternative therapeutic strategies [8].

Herein, we report a novel case of CML with a rare four-way variant Ph translocation accompanied by a cryptic double Ph chromosome, which highlights both the diagnostic challenges and the importance of advanced cytogenetic analysis in guiding targeted therapy.

## Case presentation

A 73-year-old male with a history of diabetes mellitus, Graves' disease, and interstitial lung disease was referred due to an elevated white blood cell count (WBC 32,100/μL; neutrophils 59.0%, lymphocytes 8.5%, monocytes 2.0%, basophils 15.0%, eosinophils 14.0%) detected during routine endocrinology follow-up. The patient was asymptomatic with no fever, lymphadenopathy, or splenomegaly. Peripheral blood analysis results are shown in Table 1.

**Table 1:** Transition of peripheral blood analysis.

	Reference range	Six months before diagnosis	At diagnosis	One month after dasatinib initiation
White blood cell	4000-9000/μL	9100	32,100	3400
Neutrophil	40-60%	61.9	59.0	78.7
Lymphocyte	20-40%	21.9	8.5	10.7
Monocyte	0-7%	4.5	2.0	7.7
Basophil	0-2%	4.9	15.0	0.3
Eosinophil	0-5%	7.7	14.0	0.3
Red blood cell	400-550 × 10^4^/μL	462	473	422
Hemoglobin	13.0-17.0 g/dL	11.4	13.2	11.6
Platelet	15.0-35.0 × 10^4^/μL	34.9	59.9	11.5
Aspartate aminotransferase	13-30 IU/L	9	18	146
Alanine aminotransferase	10-42 IU/L	7	24	77
Lactate dehydrogenase	135-225 IU/L	148	231	720
Total bilirubin	0.2-1.2 mg/dL	1.0	0.9	2.1
Albumin	4.1-5.1 g/dL	3.5	4.2	3.7
Blood urea nitrogen	8-20 mg/dL	11.9	11.3	23.6
Creatinine	0.65-1.07 mg/dL	0.44	0.40	0.95
Sodium	138-146 mEq/L	134	134	142
Potassium	3.6-4.9 mEq/L	4.8	4.9	4.2
Chlorine	99-109 mEq/L	99	98	101
Calcium	8.8-10.1 mg/dL	-	9.2	8.7

**Figure 1:**
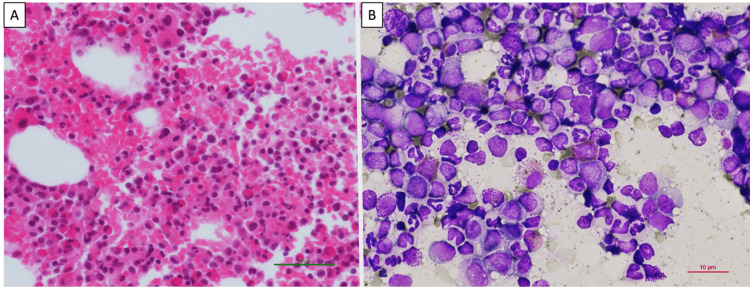
Bone marrow specimen at diagnosis of chronic myeloid leukemia. Hematoxylin-eosin staining (A) showed hypercellular marrow with marked myeloid hyperplasia. There is an increase in granulocytic precursors, including neutrophils, band forms, myelocytes, and metamyelocytes, with predominantly mature granulocytes present in May-Giemsa staining (B).

**Figure 2:**
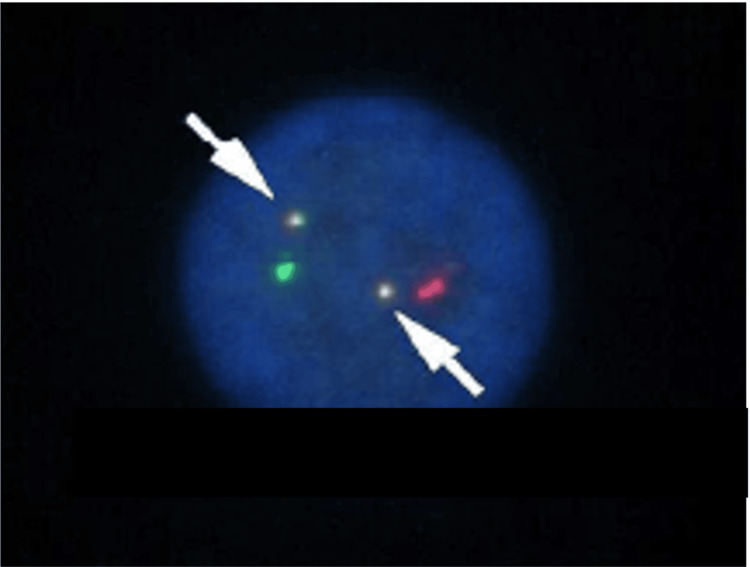
Fluorescence in situ hybridization analysis of bone marrow aspirate at diagnosis of chronic myeloid leukemia. Fluorescence in situ hybridization analysis demonstrated positive signals for theBCR::ABL fusion gene. Specifically, double dual-fusion signals (arrowhead), indicative of BCR::ABL gene rearrangement, were detected in 98% of interphase nuclei examined. The green probe indicates BCR (22q11.2) and the red probe indicates ASS1 (9q34.11) andABL (9q34.1).

Bone marrow aspiration showed moderately hypercellular marrow with markedly increased mature myeloid cells, consistent with CML (Figure 1A) and 2% of myeloblasts were identified in the smear specimen (Figure 1B). The BCR::ABL fusion transcript was significantly elevated (73.78% International Scale) in peripheral blood, and fluorescence in situ hybridization (FISH) revealed double BCR::ABL fusion signals in bone marrow (Figure 2).

**Figure 3:**
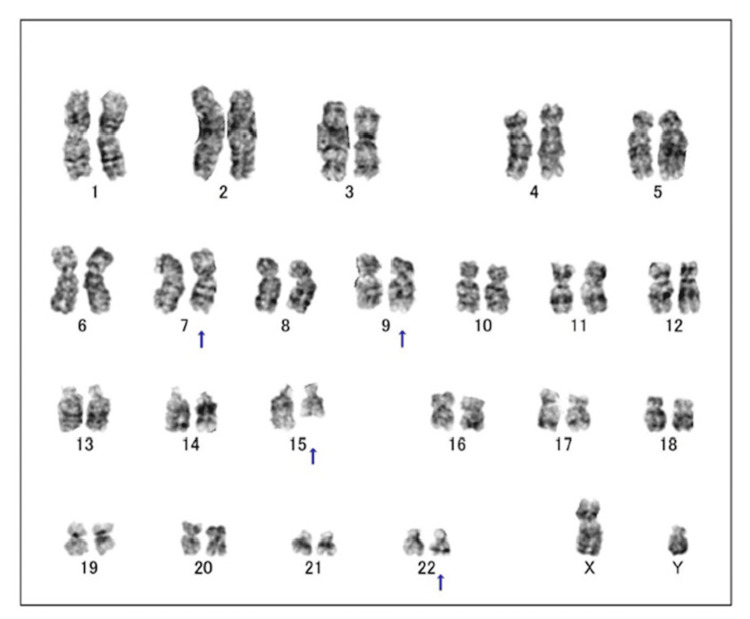
G-band karyotyping at the diagnosis of the patient. The arrowhead indicates the chromosomal abnormality, implying t(7;22;9;15)(p15;q11.2;q34.1;q22).

**Figure 4:**
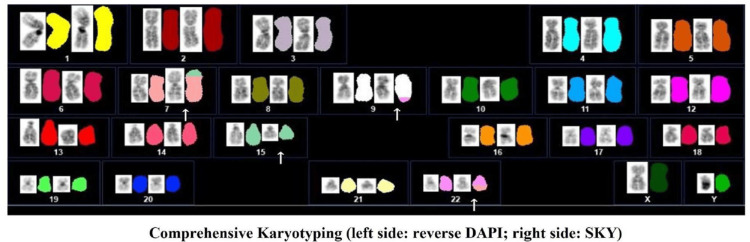
Spectral karyotyping at the diagnosis of the patient The arrowhead indicates the chromosomal abnormality, confirming t(7;22;9;15)(p15;q11.2;q34.1;q22). DAPI: 4',6-diamidino-2-phenylindole, SKY: spectral karyotyping

G-band karyotyping of the patient implied a four-way translocation involving chromosomes 7, 9, 15, and 22, but the exact chromosome structure could not be determined. It was thus described according to an International System for Human Cytogenomic Nomenclature [9] as: 46, XY, t(7;22;9;15)(p15;q11.2;q34.1;q22) (Figure 3). Therefore, we performed spectral karyotyping (SKY), which successfully identified a translocation involving chromosomes 7, 9, 15, and 22, with a potential partial deletion of chromosome 15, finally interpreted as 46, XY, t(7;22;9;15)(p15;q11.2;q34.1;q22) (Figure 4).

Neither G-band nor SKY was able to detect the double Ph chromosome evident by FISH. According to the ELN 2020 criteria [10], the disease might be categorized as AP due to the presence of double Ph (possible second Ph); however, the WHO 2022 classification [11] considered it CP CML, as AP is no longer recognized in the current edition. Given the patient's advanced age and the presence of variant and double Ph chromosomes, dasatinib (DAS) therapy (50 mg/day) was initiated [12]. One month later, the patient achieved a complete hematologic response (CHR) without significant side effects (Table 1). The patient maintained CHR for approximately three months after starting dasatinib therapy. Subsequently, he contracted COVID-19, during which CHR was still confirmed. Unfortunately, the patient later passed away due to COVID-19-related complications, and long-term therapeutic outcomes could not be further assessed.

## Discussion

The rarity of simultaneous four-way variant Ph translocation and double Ph chromosomes poses diagnostic and therapeutic challenges. Here, we discuss the diagnosis and treatment of CML harboring variant Ph and double Ph.

Four-way translocation

Frequently involved chromosomes in reported four-way translocations, besides chromosomes 9q34 and 22q11, include 1p36, 3p21, 5q13, 6p21, 11q13, 12p13, and 17p13 [4]. This case is novel in that it involves a previously unreported translocation between 7p15 and 15q22. Although there is no established treatment specifically for four-way translocation cases, TKI therapy has been reported to achieve outcomes comparable to those in standard CML [4]. Therefore, we selected DAS as the TKI therapy for this patient, and CHR was achieved.

Cryptic double Ph

The cryptic double Ph chromosome in our case is notable since small chromosomal abnormalities are often overlooked in G-banding analyses [13]. Although SKY is typically employed to identify cryptic Ph chromosomes, limitations exist due to optical resolution, image processing challenges, and probe coverage issues, especially for fragments below 1 Mb or repetitive centromeric regions [14-15]. The failure of G-band and SKY to detect the cryptic double Ph in our case suggests that only FISH might reliably identify these small, complex derivatives. Thus, the limitation in detecting cryptic Ph chromosomes by conventional cytogenetics (G-band, SKY) underscores the essential role of FISH in complex variant translocations. Although our patient achieved a rapid CHR following DAS therapy, cases with concurrent four-way variant Ph translocations and double Ph chromosomes are exceedingly rare, with only a few documented reports available in the literature. Table 2 summarizes previously reported cases of variant Ph translocations accompanied by double Ph chromosomes [16-19].

**Table 2:** Reported cases of variant Ph translocations accompanied by double Ph chromosomes. AP: accelerated phase, BC: blast crisis, CP: chronic phase, DAS: dasatinib, HSCT: hematopoietic stem cell transplantation, IMA: imatinib, MR: minor response.

Case	Cytogenetics	Variant Ph	Clinical stage at diagnosis	Treatment/outcome	Course/outcome
Al‑Achkar et al. 2013 [16]	45,XX,der(7)t(7;8)(p11.2;q11.2),‑8,der(9)(20qter→20q11.2::9p21→9q34::22q11.2→22qter),‑12,der(12) (20pter→20q11.2::12p13.3→12q24.3::12q24.3→12q15~21.1),+der(22)t(9;22)(q34;q11.2)x2/45,XX, der(7)t(7;8) (p11.2;q11.2),‑8,t(9;22)(q34;q11.2)	Four-way translocation	BC	IMA/unknown	Died for unknown reason under treatment
Li et al. 2018 [17]	46, XY,idicder(22) t(3;9;22)(p21;q34;q11)/46, XY, t(9;22)(q34;q11)	Three-way translocation	BC	IMA, DAS, Allo-HSCT/CMR	Alive
Aliano et al. 2013 [18]	46,XY,t(9;11;22)(q34;p15;q11)	Three-way translocation	AP	IMA, Allo-HSCT/MR→relapse	Died for blast crisis
Sheth et al. 2005 [19]	47,XY,t(4;9;22)(q25;q34;q11.2),der(22)del(22)(q11),Ph+,+mar.	Three-way translocation	CP	Interferon-α, chemotherapy/unknown	Alive for three to five years

Most of these cases presented in advanced phases (AP or BC) at diagnosis or progressed shortly thereafter, demonstrating prominent resistance to first-generation TKI, and thus necessitating treatment with second-generation TKIs, chemotherapy, or allogeneic hematopoietic stem cell transplantation. As demonstrated in our case, if the disease is classified as CP CML according to the WHO 2022 criteria, second-generation TKI therapy might lead to favorable treatment outcomes. Given the rarity of these cases, however, accumulating and analyzing similar cases is critical for developing effective treatment strategies.

As a limitation, the follow-up period was relatively short, since the patient developed COVID-19 three months after treatment initiation and subsequently died due to COVID-19-related complications. Although a complete hematologic response was maintained until that time, the long-term efficacy of dasatinib could not be assessed. Therefore, further follow-up data and additional similar cases will be necessary to better understand the clinical course of CML with such complex cytogenetic abnormalities.

## Conclusions

In conclusion, we describe a rare case of chronic-phase CML with both a novel four-way variant Philadelphia translocation and cryptic double Philadelphia chromosomes. This unusual combination of abnormalities posed diagnostic challenges, as conventional cytogenetic methods failed to detect the additional Ph chromosome, underscoring the indispensable role of FISH in identifying cryptic chromosomal changes. Despite the presence of these unfavorable cytogenetic features, the patient achieved a complete hematologic response with dasatinib, demonstrating that second-generation TKIs can be effective even in atypical and high-risk settings. This case emphasizes the importance of comprehensive cytogenetic evaluation in guiding therapeutic decisions and highlights the need for further accumulation of similar cases to better understand the clinical implications and optimize management strategies for rare cytogenetic variants of CML.
